# Isolation and Molecular Characterization of *Mycoplasma gallisepticum* and *Mycoplasma synoviae* in Chickens in Sudan

**DOI:** 10.1155/2013/208026

**Published:** 2013-09-18

**Authors:** Khalda A. Khalifa, Egbal Sidahmed Abdelrahim, Magdi Badwi, Amal M. Mohamed

**Affiliations:** Veterinary Research Institute (VRI), P.O. Box 806, Khartoum, Sudan

## Abstract

The current study described the isolation and molecular detection of *Mycoplasma gallisepticum* (Mg) and *Mycoplasma synoviae* from tracheal swabs of diseased birds showing signs of respiratory distress in selected commercial (layer and broiler) farms and from yolk and an open air of pens of vaccinated breeder flocks in Sudan. A number of 45 *Mycoplasma* isolates were recovered from chickens in Khartoum, Gezira, and Equatoria states in Sudan. Of these, eight Mg and three Ms isolates were identified using growth inhibition and rapid serum agglutination (RSA) tests. The conventional PCR technique was applied to amplify 140 bp and 720 bp DNA fragments for the Mg and Ms, respectively. This research confirmed vertical and horizontal transmission of Mg from breeder farms through detection of Mg in yolk of fertile eggs and an air of pens despite previous vaccination. PCR is considered a rapid, sensitive, and cheap method and it will improve the diagnosis of *Mycoplasma* in chickens.

## 1. Introduction

Avian mycoplasmosis was primarily described in turkeys in 1926 and in chickens in 1936 [1]. *Mycoplasma gallisepticum* (Mg) infection is usually designated as chronic respiratory disease of chickens and infectious sinusitis in turkeys. It is characterized by respiratory rales, coughing, nasal discharges, and frequently by sinusitis in turkeys by synovitis. *Mycoplasma synoviae* (Ms) infection is usually known as infectious synovitis, an acute-to-chronic infectious disease for chickens and turkeys involving primarily the synovial membranes of joints and tendons sheaths. However, during recent years, Ms has less frequently been associated with synovitis but more frequently associated with airsacculitis in chickens and sometimes in turkeys [2]. Both diseases are economically important, egg transmitted and hatchery disseminated diseases. They lead to tremendous economic losses in poultry production as a result of decreased hatchability and egg production, reduced quality of day-old chicks, reduced growth rate, increased costs of eradication procedures which involve site cleaning and depopulation, and increased costs of medication and vaccination [3].

The first isolation of both mycoplasmas in Sudan was reported by Khalda [4]. A recent study indicated that these organisms were prevalent, as 50.8% Mg and 57.6% Ms antibodies were recorded in chickens in the country [5].

For many years, diagnosis of avian mycoplasmosis was based on serological assays to detect antibody production and/or on isolation and identification of the organism. Serological tests include the rapid slide agglutination test, the haemagglutination inhibition test, and ELISA for Mg, Ms, or *M. meleagridis *[6, 7]. Cultivation techniques are laborious, slow, and expensive and require sterile conditions. Problems experienced with culture include overgrowth by faster-growing *Mycoplasma* species or other organisms, or no growth in subculture. Particularly in difficult cases, *in vivo *bioassays are necessary and involve the inoculation of specific pathogen-free chickens with suspect material [7].

Saiki et al. [8] developed new techniques based on DNA amplification. Consequently, different many PCR assays for detection of the avian *Mycoplasma* have been reported [9–13].

The aim of this study is to detect Mg and Ms in chickens in Sudan using molecular tools.

## 2. Materials and Methods

### 2.1. Samples for *Mycoplasma* Isolation

The source of samples was from different farms in Khartoum, Gezira and Equatoria states which were submitted for diagnosis to the Department of Avian Pathology and Diagnosis at the Veterinary Research Institute, Khartoum during the year 2005-2006. A total of 170 tracheal swabs were collected from diseased birds. From each farm, 4-5 swabs were pooled compromising (16 from Khartoum, 11 from Gezira and 8 from Equatoria states) in addition and five swabs each collected from air and egg yolk of a breeder farm in Khartoum state that had a history of Mg vaccination with a commercial killed vaccine. The diseased birds showed rales, swollen face and eyes with lacrimation, cyanotic comb and wattles, nasal discharge, thin egg shell, and drop in production. The collected swabs were kept in 20°C until being cultured to isolate the *Mycoplasma*. 

### 2.2. Cultural Method

 Pooled swabs from each farm were streaked on PPLO (pleuro-pneumonia-like organism) agar plates, incubated for 7 days at 37°C as described [14]. When the growth of the colonies was obtained, digitonin test was performed to differentiate the colonies of *Mycoplasma* from *Acholeplasma*. Colning was done using broth culture, identification of Mg and Ms was made by the growth inhibition test using specific antisera (BioChek) as described [15] and the rapid serum agglutination tests for the two species. Then, positive cultures were lyophilized and kept in −20°C to be used for DNA extraction. 

Positive samples were investigated by PCR method. The details of these samples were shown in Table 1.

### 2.3. Molecular Methods

#### 2.3.1. DNA Extraction

DNA was extracted using a commercial kit (Sacace Biotechnology, Italy) according to the kit manufacturer's protocol. The final volume of the extracted DNA was 50 *μ*L.

#### 2.3.2. DNA Amplification

Amplification was performed following the kit manufacturer's instructions. The number of readymade PCR mix1 tubes which contain primers was defined according to the number of samples used. A volume of 10 *μ*L of PCR mix2 plus 10 *μ*L of template sample were mixed together and added to PCR mix1. Ten *μ*L of DNA buffer and 10 *μ*L of the provided positive control were used in the test as negative and positive controls, respectively.

All these tubes were put in thermocycler “PTC-100” (MJ Research) from BioRad, Biometra for amplification. The reaction mixtures were subjected to 43 cycles. Each one involved the following cyclic profile: denaturation at 95°C for 2 min, primer annealing at 95°C for 1 min, 61°C for 1 min, and 72°C for 1 min. Extension at 72°C for 1 min; then, the PCR products were stored at 10°C till electrophoresis.

#### 2.3.3. Electrophoresis

A volume of 5 *μ*L of loading buffer solution was added to 10 *μ*L of PCR product and analyzed by electrophoresis in 2% agarose gel in TBE buffer then stained with 0.5 *μ*g ethidium bromide and visualized under UV light using image master (VDS Pharmacia Biotech). 100 bp DNA marker was used (Vivantis, Malaysia).

## 3. Results

### 3.1. Culture of Swabs

Colonies of fried egg appearance on solid media were observed in all cultures. All the colonies were found sensitive to digitonin, insuring that they were mycoplasmas.

### 3.2. Culture Identification

 Seven isolates (4 Mg and 3 Ms) were found positive by growth inhibition test and two Mg isolates by the rapid agglutination test.

### 3.3. Results of DNA Amplification

**Figure 1:**
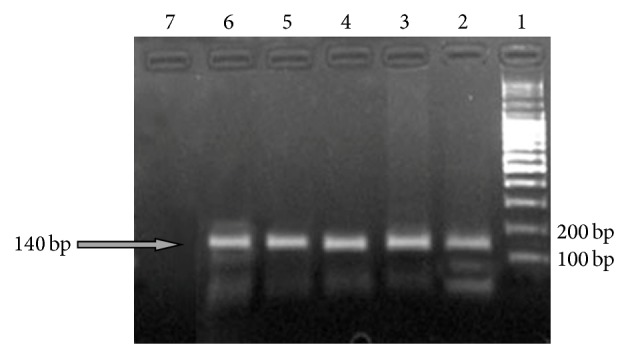
Electrophoresis profile of Mg DNA obtained from 5 samples by PCR (140 bp) on 2% Agarose Gel. Lane 1: DNA Ladder weight marker; Lane 2: isolate A; Lane 3: isolate B; Lane 4: isolate C; Lane 5: isolate D, Lane 6: positive control; Lane 7: negative control.

**Figure 2:**
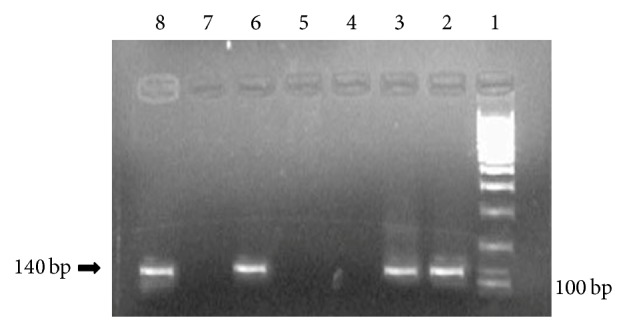
Electorophoresis profile of Mg DNA obtained from 4 samples by PCR (140 bp) on 2% Agarose Gel. Lane 1: DNA Ladder; Lane 2: isolate E; Lane 3: isolate G; Lane 4: negative sample; Lane 5: negative sample; Lane 6: isolate H; Lane 7: negative control; Lane 8: isolate I.

Eight isolates A, B, C, D, E, G, H, and I of pooled 22 swabs (17.8%) of 45 samples were found positive to Mg (Table 2) as they gave 140 bp products, similar to the positive control when visualized electrophorically (Figures 1 and 2). 

**Figure 3:**
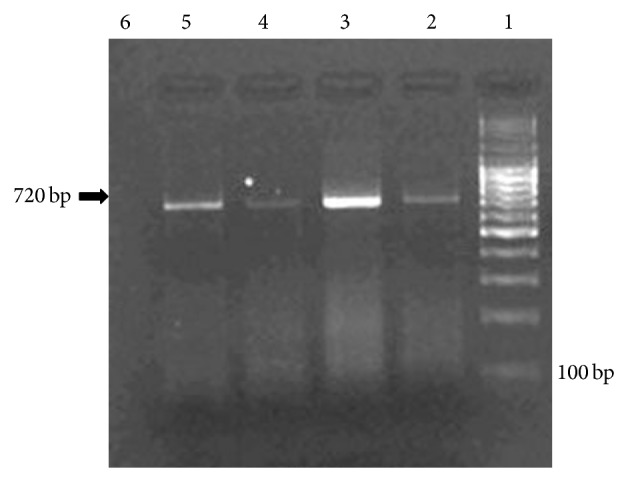
Electorophoresis profile of Ms DNA obtained from 3 samples by PCR (740 bp) on 2% Agarose. Lane 1, DNA Ladder; Lane 2: isolate F; Lane 3: J; Lane 4: K; Lane 5, positive control; Lane 6: negative control.

The three remaining isolates (F, J and K) representing 4 swabs (6.7%) were found positive to Ms (Table 2) as 720 bp products were obtained similar to the positive control of the kit, (Figure 3).

## 4. Discussion

In this study, Mg and Ms were detected in chicken in selected farms of breeder, broilers, and layers flocks of different ages which confirmed that there was a wide age susceptibility to these organisms. Both organisms were isolated and PCR detected in local chickens. This finding was consistent with the previous study [5] is revealed 21.6%, 71.6% Mg and Ms antibodies, respectively, in local chickens in Sudan. These results raise the attention to the role of this breed in the transmission of the disease; moreover, it necessitates the importance of treatment and prophylactic programme of this breed. It was noticed that some of the studied flocks were affected with avian influenza which shares the same clinical signs of both mycoplasmas; this mixed infection will impose to put in consideration *Mycoplasma* infection in such cases with respiratory distress. 

The present investigation proved vertical and horizontal transmissions of Mg from breeder flocks through detection of it in the egg yolk of fertile eggs and open air. This was in agreement with [12] which detected Mg by PCR technique in pipped embryos, normal chicks, and breeder flocks in Malaysia. The vertical transmission is very important as Mg can be passed to the embryo and it may affect its hatchability. Furthermore, if the infected progenies are introduced into flocks they later may serve as source of horizontal infection [12]. The presence of Mg in vaccinated breeder flocks in the current research may show the failure of the Mg eradication program or it may show as previously speculated [12, 16] that inactivated bacterin is an important part of control program, but it provides minimal protection. Challenged vaccinates may be infected with pathogenic Mg thus egg transmission and lateral spread still occur, [17].

Since 1970, the diagnosis of Mg and Ms in Sudan was made by the demonstration of antibodies against both of them by the serum agglutination test. However, Khalda [4] was the first to isolate and confirm Mg and Ms by growth inhibition test from Sudan. In this research, PCR technique is applied for the detection of Mg and Ms in different samples which is considered as sensitive and rapid tool. The cost of PCR can be reduced by pooling samples as previously reported [12]. PCR method can improve the diagnosis of *Mycoplasma* in Sudan as the conventional isolation is time consumable in addition, it can be used in epidemiology and control of Mg and Ms infections and decontamination of farm and hatcheries.

To the best of our knowledge, this is the first report on molecular identification of Mg and Ms in Sudan. More researches should be conducted for further serotyping of these isolates, using modern technique such as restriction enzyme analysis is recommended.

## Figures and Tables

**Table 1:** Labels of positive Mg and Ms culture farms, type, breed, and age of chickens.

State	Locality	Label	No. and type of positive pooled samples (swabs)	Type of bird production	Breed	Age
Khartoum	Soba	A	5 air	Breeder	Bovan	1 year
	Soba	B	5 yolk	Breeder	Bovan	1 year
	Geraif	C	3 tracheal	Layer	Lohman	4 months
	Geraif	D	1 tracheal	Layer	Bovan	6 months
	Bagair	E	2 tracheal	Broiler	Hypro	42 day
	Dekhainat	F	2 tracheal	Layer	Lohman	1 year
	Kabashi	G	4 tracheal	Layer	Hisex	4 months

Gezira	Medani	H	1 tracheal	Layer	Hyline	4 months

Equatoria	Lado hill	I	1 tracheal	Layer	Local	Adult
	Lado hill	J	1 tracheal	Layer	Local	Adult
	Lado hill	K	1 tracheal	Layer	Local	Adult

Total			26			

**Table 2:** Result of Mg and Ms isolates identified by GIT and RSA tests and PCR assay.

State	Locality	Label	Primary test	Mg PCR	Ms PCR
Khartoum	Soba	A	GIT	+ve	−ve
	Soba	B	GIT	+ve	−ve
	Geraif	C	Not tested	+ve	−ve
	Geraif	D	GIT	+ve	−ve
	Bagair	E	RSA	+ve	−ve
	Dekhainat	F	GIT	−ve	+ve
	Kabashi	G	RSA	+ve	−ve

Gezira	Medani	H	GIT	+ve	−ve

Equatoria	Lado hill	I	Not tested	+ve	−ve
	Lado hill	J	GIT	−ve	+ve
	Lado hill	K	GIT	−ve	+ve

Total				8 +ve	3 +ve

GIT: growth inhibition test; RSA: rapid serum agglutination test.
